# Connexin26 gap junction mediates miRNA intercellular genetic communication in the cochlea and is required for inner ear development

**DOI:** 10.1038/srep15647

**Published:** 2015-10-22

**Authors:** Yan Zhu, Liang Zong, Ling Mei, Hong-Bo Zhao

**Affiliations:** 1 Department of Otolaryngology, University of Kentucky Medical Center, 800 Rose Street, Lexington, KY 40536

## Abstract

Organ development requires well-established intercellular communication to coordinate cell proliferations and differentiations. MicroRNAs (miRNAs) are small, non-coding RNAs that can broadly regulate gene expression and play a critical role in the organ development. In this study, we found that miRNAs could pass through gap junctions between native cochlear supporting cells to play a role in the cochlear development. Connexin26 (Cx26) and Cx30 are predominant isoforms and co-express in the cochlea. Cx26 deficiency but not Cx30 deficiency can cause cochlear developmental disorders. We found that associated with Cx26 deletion induced the cochlear developmental disorders, deletion of Cx26 but not Cx30 disrupted miRNA intercellular transfer in the cochlea, although inner ear gap junctions still retained permeability after deletion of Cx26. Moreover, we found that deletion of Cx26 but not Cx30 reduced miR-96 expression in the cochlea during postnatal development. The reduction is associated with the cochlear tunnel developmental disorder in Cx26 knockout (KO) mice. These data reveal that Cx26-mediated intercellular communication is required for cochlear development and that deficiency of Cx26 can impair miRNA-mediated intercellular genetic communication in the cochlea, which may lead to cochlear developmental disorders and eventually congenital deafness as previously reported.

Tissue homeostasis and organ development rely on the well-orchestrated integration of intercellular communication and gene regulation to synchronize and coordinate cell proliferation and differentiation^1^. Gap junctions are intercellular channels and represent the only selective intercellular conduit that possesses a large pore size (1.0–1.5 nm), allowing direct exchange of ions and small molecules between cells^2^. It has been reported that small regulatory RNAs, such as siRNAs and miRNAs, can also pass through gap junctions^3^,^4^,^5^,^6^,^7^,^8^,^9^,^10^, which provides a novel mechanism for intercellular genetic communication^11^. In particular, miRNAs are single-stranded RNAs consisting of ~21 nucleotides and can broadly modulate gene expression by affecting the translation of mRNAs to proteins and mRNA target decay^12^,^13^,^14^,^15^. To date, approximately 300 conserved miRNA families and thousands of additional poorly conserved miRNAs have been identified in mammals. Approximately two thirds of all human protein-coding genes are conserved targets of miRNAs^13^,^14^. Thus, miRNAs provide a widespread mechanism for post-transcriptional control of gene expression and are important for the organ development.

Gap junctions have a crucial role in hearing. Connexin26 (Cx26, *GJB2*) mutations cause most cases of hereditary genetic deafness, responsible for >50% of nonsyndromic hearing loss^16^,^17^. Recently, we found that Cx26 deficiency can cause cochlear developmental disorders leading to congenital deafness^18^,^19^. However, the underling mechanism for developmental disorders remains unclear. In this study, we found that miRNAs can pass through gap junctions in the cochlea. Cx26 and Cx30 are predominant connexin isoforms in the cochlea^20^,^21^. Associated with Cx26 deficiency induced cochlear developmental disorders, Cx26 deficiency but not Cx30 deletion disrupted miRNA-mediated intercellular genetic communication in the cochlea.

## Results

### Gap junction and Cx26 and Cx30 expression in the cochlea

**Figure 1:**
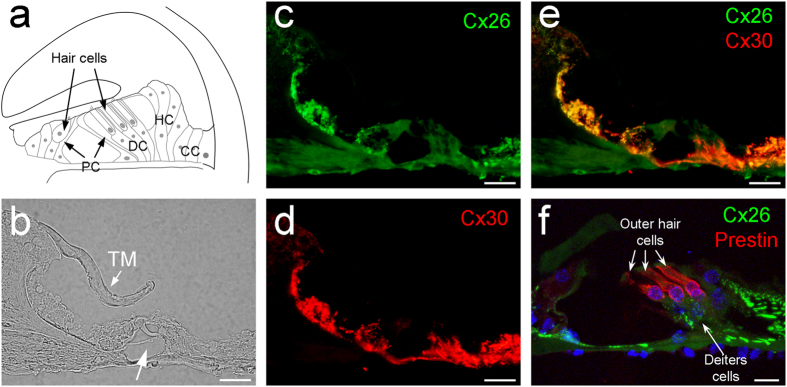
Cochlear structure and co-expression of Cx26 and Cx30 in the cochlea. (**a**) Schematic drawing of the cochlear structure in the cross-section. PC: Pillar cell, DC: Deiters cell, HC: Hensen cell, CC: Claudius cell. (**b–e**) Immunofluorescent staining for Cx26 (green) and Cx30 (red) in the cochlea. A white arrow in panel (**b**) indicates the cochlear tunnel. TM: tectorial membrane. (**f**) A high-magnitude image in the organ of Corti. Outer hair cells were visualized by prestin labeling (red). Scale bars: 25 μm in (**b**–**e**), 10 μm in (**f**).

The organ of Corti has hair cells and supporting cells (Fig. 1a). The auditory sensory hair cells have no gap junctional coupling and connexin expression (Fig. 1f and also see ref. 20, 21). Gap junctions and connexin expression only existed in supporting cells (Fig. 1c–f). The organ of Corti contains four types of supporting cells, i.e., Deiters cells (DC), pillar cells (PC), Hensen cells (HC), and Claudius cells (CC) (see Supplementary Fig. S1). All of them had Cx26 and Cx30 expression and were well-coupled (Fig. 1 and also see ref. 20,21).

### Blockage of miRNA intercellular transfer by gap junctional blockers

The intercellular transfer of miRNAs between cochlear supporting cells could be blocked by gap junctional blockers. Fig. 3 shows that application of 50 μM 18α-glycyrrhetinic acid (18-AGA) or 0.1 mM carbenoxolone (CBX) gap junctional blockers blocked miR-F diffusion between cells. The injected miR-F nucleotides were restricted within the injected cell and did not diffuse into the adjacently-contacted cells. Gap junction blocker blocked not only miR-F diffusion but also dye ethidium bromide (EB) diffusion between cells (Fig. 3d).

### Cochlear development disorders and disruption of miRNA intercellular transfer in Cx26 KO mice

As previously reported^18^,^22^, deletion of Cx26 could induce cochlear developmental disorders (Fig. 4). The tectorial membrane was attached to the inner sulcus cells and the cochlear tunnel was filled (Fig. 4e). Deletion of Cx26 also disrupted intercellular transfer of miRNAs in the cochlea (Fig. 5). The injected miR-F was restricted to the injected cell (Fig. 5c–f). In all 12 injections, no intercellular diffusion of miR-F was visible. However, deletion of Cx26 did not completely disrupt inner ear gap junctions, which still retained permeability to dye EB (Fig. 5g,h). Input capacitance (*C*_*in*_) recording also indicated that cochlear supporting cells in Cx26 KO mice still retained good gap junctional coupling. *C*_*in*_ in the recording Hensen cells in Fig. 5e,f and Claudius cells in Fig. 5g,h was ~75 pF and 19.3 pF, respectively, showing that they were well-coupled.

### Normal cochlear development and miRNA intercellular transfer in Cx30 KO mice

Cx30 is co-expressed with Cx26 in the cochlea (Fig. 1, and also see ref. 20, 21). However, deletion of co-expressed Cx30 displayed normal cochlear development (Fig. 6a–b). Intercellular transfer of miR-F also appeared normal in Cx30 KO mice and intercellular diffusion of miR-F among supporting cells was visible (Fig. 6c–f).

### Reduction of miRNA expression in Cx26 KO mice during cochlear postnatal development

MicroRNA-96 is critical for cochlear development^23^. In mouse postnatal development, the cochlear tunnel starts to open at postnatal day 5 (P5) and fully opens at P10 (Fig. 7a). We found that prior to the cochlear tunnel opening, the expression of miR-96 in the cochlea was increased at P3 (Fig. 7b). Then, the expression decreased and reached a steady state at P10. However, the expression of miR-96 in Cx26 KO mice was not increased at P3 and remained at lower level during the postnatal period (Fig. 7b). On the other hand, the expression of miR-96 in the cochlea in Cx30 KO mice, which displayed normal cochlear development (Fig. 6a), was similar to WT mice, increasing at P3 and then reducing afterward during the postnatal development (Fig. 7b). There was no significant difference in miR-96 expression between Cx30 KO mice and WT mice (P = 0.43, one-way ANOVA).

## Discussion

**Figure 2:**
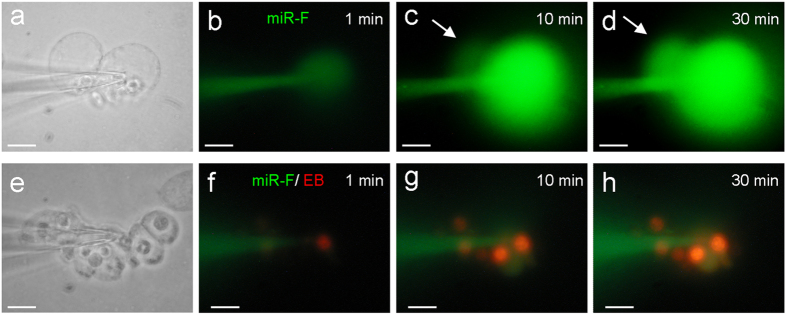
Time-lapse recording of intercellular transfer of miR-F between cochlear supporting cells. (**a–d**) Diffusion of miR-F between Hensen cells. The miR-F nucleotides were injected into a Hensen cell by the patch pipette. Arrows indicate a neighboring cell with miR-F labeling. (**e–h**) Intercellular diffusion of miR-F and EB between Claudius cells. The patch pipette was filled with a mixture of miR-F and EB. EB labeling is mainly visible at cell nuclei because of EB binding to DNAs. Scale bars: 10 μm.

**Figure 3:**
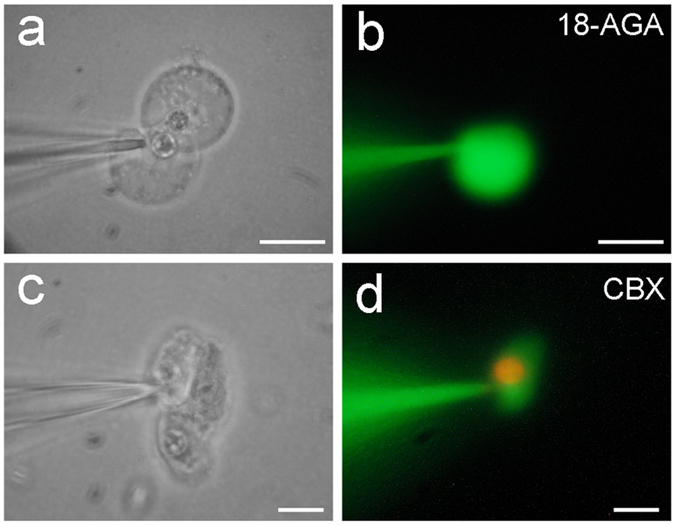
Blockage of miRNA and EB diffusion between the cochlear supporting cells by gap junctional blockers. (**a**,**b**) Blockage of intercellular diffusion of miR-F between Hensen cells by application of 50 μM 18-AGA. (**c**,**d**) Intercellular difussion of miR-F and EB between Claudius cells was blocked by application of 0.1 mM CBX. All images were captured after injection for 30 min. Scale bars: 10 μm.

**Figure 4:**
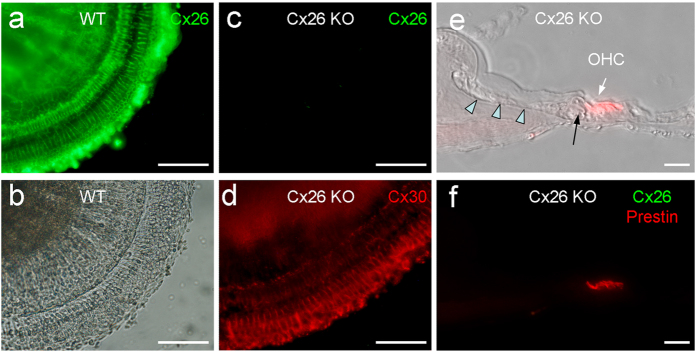
Cx26 deletion in the cochlear sensory epithelium and developmental disorders in Cx26 KO mice. (**a**,**b**) Immunofluorescent staining for Cx26 in the cochlear sensory epithelium of WT mice. (**c**,**d**) Immunofluorescent staining for Cx26 (green) and Cx30 (red) of the cochlear sensory epithelium in the Cx26 KO mice. No Cx26 labeling is visible but Cx30 labeling remains. (**e**,**f**) Cochlear developmental disorders in Cx26 KO mice. The tectorial-membrane attaches to the inner sulcus cells (indicated by arrow heads) and the cochlear tunnel is filled (indicated by a black arrow). Outer hair cells (OHCs) are visualized by prestin labeling (red). Immunofluorescent staining for Cx26 (green) is negative. Scale bars: 100 μm in (**a**–**d**), 25 μm in (**e**,**f**).

**Figure 5:**
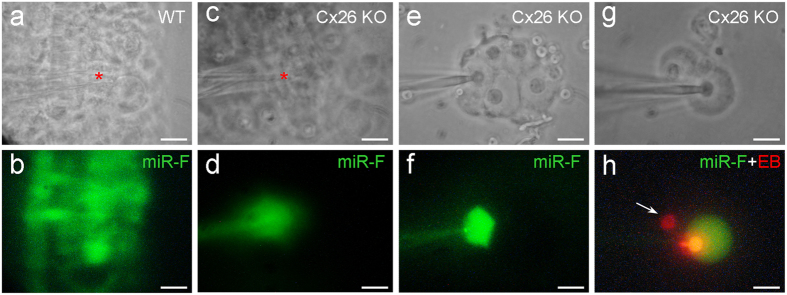
Disruption of the intercellular transfer of miRNAs between cochlear supporting cells in Cx26 KO mice. (**a**,**b**) Intercellular transfer of miR-F in the mouse cochlear sensory epithelium. The injection site (indicated by a red asterisk in panel (**a**)) locates at the Hensen cell region in the cochlear sensory epithelium. (**c**,**d**) Disruption of miR-F intercellular transfer in the cochlear sensory epithelium in Cx26 KO mice. A red asterisk in panel **c** indicates the injection site, where locates at the Hensen cell region. (**e**,**f**) Disruption of intercellular transfer of miRNA between cochlear supporting cells in Cx26 KO mice in the isolated cell preparation. The injected miR-F is limited in the injected Hensen cell. *C*_*in*_ is ~75 pF, indicating that these cells are well-coupled by gap junctions. (**g**,**h**) Disruption of intercellular diffusion of miR-F but not EB between cochlear supporting cells. The pipette was filled with a mixture of miR-F and EB. An arrow indicates that a neighboring Claudius cell only has red EB labeling but no miR-F labeling. *C*_*in*_ is 19.3 pF, indicating that two cells are coupled. All images were captured after injection for 30 min. Scale bars: 25 μm in (**a**–**d**), 10 μm in (**e**–**h**).

**Figure 6:**
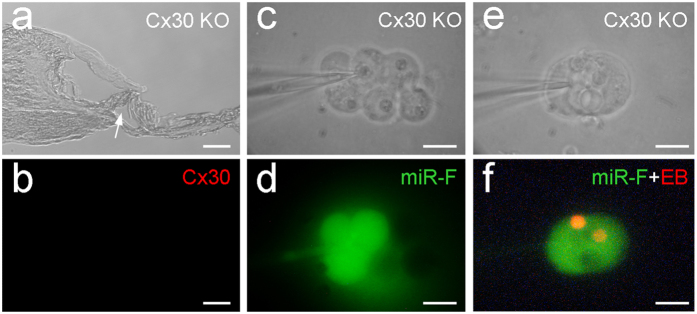
Normal cochlear development and miRNA intercellular transfer in the cochlea in Cx30 KO mice. (**a**–**b**) Normal cochlear development in the Cx30 KO mice. A white arrow in panel (**a**) indicates the open cochlear tunnel. Panel (**b**) shows immunofluorescent staining for Cx30. No Cx30 labeling is visible. (**c**–**d**) Intercellular transfer of miR-F between cochlear supporting cells in Cx30 KO mice. *C*_*in*_ = 43 pF. (**e**–**f**) Intercellular transfer of miR-F and EB between a pair of Hensen cells in Cx30 KO mice. *C*_*in*_ = 35.5 pF. All images were captured after injection for 30 min. Scale bars: 25 μm (**a**–**b**), 10 μm in (**c**–**f**).

**Figure 7:**
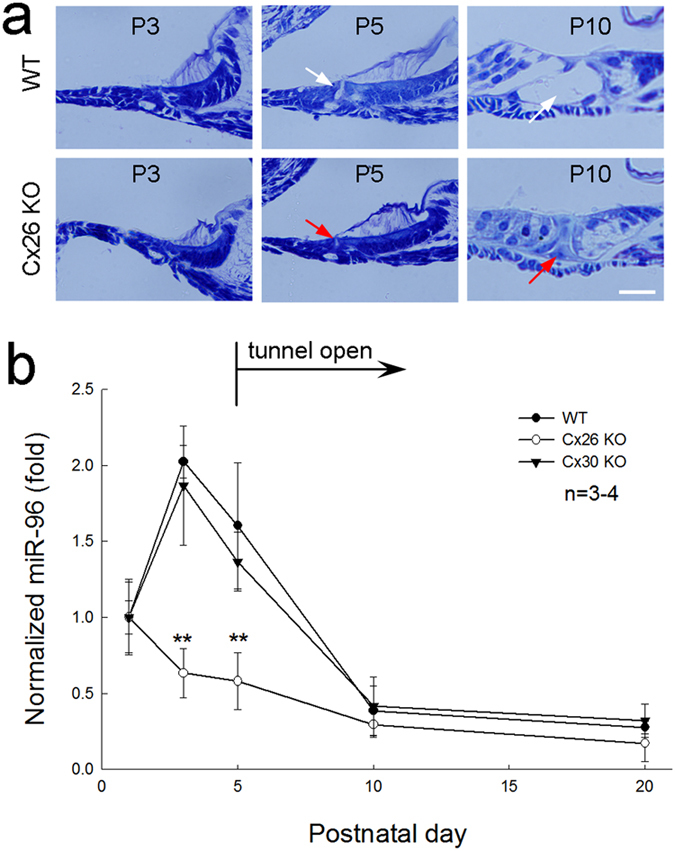
Cochlear tunnel development and expression of miR-96 in the cochlea during cochlear postnatal development. (**a**) Postnatal development of the organ of Corti in wild-type (WT) and Cx26 KO mice. The cochlear tunnel occurs at postnatal day 5 (P5) in WT mice (indicated by white arrows) but is filled in the Cx26 KO mice (indicated by red arrows). Scale bars: 20 μm. (**b**) Dynamic changes of miR-96 expression in the Cx26 KO, Cx30 KO, and WT mouse cochlea during postnatal development. WT littermates were used as control. The expression levels were normalized to that at P1 for comparison of dynamic changes. **P < 0.001, one-way ANOVA with a Bonferroni correction.

In this study, we found that miRNAs could pass through gap junctions between native cochlear supporting cells (Figs 2, 5a,b and Supplementary Fig. S2). Deletion of Cx26 disrupted cochlear development and miRNA intercellular transfer in the cochlea (Figs. 4 and 5). However, the inner ear gap junctions still remainted permeability to cationic dye EB after Cx26 deletion (Fig 5g,h). Deletion of Cx26 also reduced miR-96 expression in the cochlea during postnatal development and the reduction is associated with over-development of the cochlear tunnel (Fig. 7). However, consistent with the normal cochlear development in Cx30 KO mice, deletion of Cx30 did not affect intercellular transfer of miRNA and miR-96 expression in the cochlea (Figs 6 and 7b). We previously reported that miRNAs can pass through gap junctional channels and regulate gene expression in neighboring cells to achieve intercellular genetic communication^11^. Our new data further demonstrate that this gap junction-mediated miRNA intercellular communication may have an important role in the cochlear development.

Deletion of Cx26 can result in filling of the cochlear tunnel and attachment of the tectorial membrane to inner sulcus cells leading to loss of the under-tectorial-membrane space (Figs 4e,7a, and also see ref. 17, 18, 19,22). Currently, the underlying mechanism remains unclear. In this experiment, we found that prior to the cochlear tunnel opening, miR-96 expression had a rapid increase at P3 (Fig. 7). This peak was missed in Cx26 KO mice but not in Cx30 KO mice (Fig. 7b). We hypothesize that this up-regulation of miRNA expression may be associated with arresting of cochlear supporting cell differentiation leading to formation of the cochlear tunnel. Deletion of Cx26 impaired intercellular transfer of miRNAs between cochlear supporting cells (Fig. 5), which may lead to over-proliferation and differentiation of cochlear supporting cells inducing over-development of the cochlear tunnel and loss of the under-tectorial-membrane space. This concept is further supported by the fact that deletion of Cx26 disrupted miRNA permeability but not EB permeability in the cochlea (Fig. 5). This implies that cochlear gap junctions in Cx26 KO mice still retain permeability to ions and other small molecules, since co-expressed Cx30 expression remains (Fig. 4d, and also see ref. 24). Thus, some of gap junctional functions in the cochlea, such as K^+^-recycling, in Cx26 KO mice may still remain normal. These data also further support our previous reports that Cx26 deficiency impairs cochlear developmental disorders and active cochlear amplification rather than K^+^-recycling resulting in hearing loss^17^,^18^,^19^,^24^,^25^.

In the experiment, we found that deletion of Cx30 did not affect miRNA permeability in the cochlea (Fig. 6) and had no influence on miR-96 expression (Fig. 7b). This is consistent with our previous report that inner ear gap junctions have strong charge-selectivity and that Cx26 is mainly responsible for anionic permeability in the cochlea^26^. MicroRNAs are anionic at physiological pH. It has been found that Cx30 channels are impermeable to anionic molecules^27^,^28^ and miRNAs^11^. Thus, deletion of Cx30 could have little effect on permeability to miRNAs in the cochlea (Fig. 6). These data also suggest that Cx26 may have a critical role not only in intercellular signaling in the cochlea^26^ but also in intercellular genetic communication and development in the cochlea. This may be a reason why Cx26 rather than Cx30 deletion can induce cochlear developmental disorders.

Cx30 deficiency can also induce hearing loss^17^,^29^,^30^,^31^. However, hearing loss mainly results from endocochlear potential (EP) reduction^17^,^19^,^29^ rather than from cochlear developmental disorders as shown in Cx26 deficiency.

MicroRNAs provide a widespread mechanism for post-transcriptional control of gene expression. Gene expression can be regulated by many factors at many stages, such as enhancer and promoter, transcription factors, and mRNA polyA polymerization. However, none of these regulatory factors is intercellular-exchangeable through gap junctions except small non-coding RNAs such as miRNA. Gap junctions extensively exist in almost all cell types and organs. Recently, it has been found that miRNAs can be exchanged between tumor cells in a gap junction-dependent manner^7^,^8^,^9^. Thus, gap junction mediated intercellular genetic communication can play an important role in organ development and may also be important for tumor genesis or inhibition.

## Methods

### Cx26 KO and Cx30 KO mice and genotyping

Cx26 KO mice were generated by crossing Cx26^*loxP/loxP*^ mice (European Mouse Mutant Archive, EM00245) with the *Pax2-Cre* mouse line (the Mutation Mouse Regional Center, Chapel Hill, NC)^18^. The Cx26 floxed allele was detected on tail genomic DNA by PCR amplification using the following primers: Cx26F: 5′-CTT TCC AAT GCT GGT GGAGTG-3′ and Cx26R: 5′-ACA GAA ATG TGT TGG TGA TGG-3′^18^. Cx26^*loxP/loxP*^ and wild-type (WT) mice generated 400 and 300 bps bands, respectively. For the *Pax2-Cre* transgene, the following primers were used: CreF: 5′-GCC TGC ATT ACC GGT CGA TGC AAC GA- 3′ and CreR: 5′-GTG GCA GAT GGC GCG GCA ACA CCA TT- 3′. The band size was 700 bps. Cx30 KO mice^19^,^29^ were also purchased from EMMA (EM000323). Primer pairs for detecting Cx30 KO were Cx30 KO-1 (LACZ e Neo): 5′-GGT ACC TTC TAC TAA TTA GCT TGG -3′; Cx30 KO2 (LACZ e Neo): 5′-AGG TGG TAC CCA TTG TAG AGG AAG -3′; Cx30 KO-3 (LACZ e Neo) 5′-AGC GAG TAA CAA CCC GTC GGA TTC -3′. The bands of Cx30 KO and WT mice were 460 and 544 bps, respectively.

The experimental procedures were approved by the University of Kentucky′s Animal Care & Use Committee and conducted according to the standards of the NIH Guidelines for the Care and Use of Laboratory Animals.

### Cochlear cell isolation and intracellular injection

Adult mice (30–60 day old) were decapitated and the temporal bone was removed. As we previously reported^32^,^33^,^34^,^35^, the otic capsule was opened and the cochlea was isolated by micro-dissection in a standard extracellular solution (142 NaCl, 5.37 KCl, 1.47 MgCl_2_, 2 CaCl_2_, 10 HEPES in mM, 300 mOsm, pH 7.2). The sensory epithelium was micro-dissected by a sharpened needle. The isolated sensory epithelium was dissociated by trypsin (1 mg/ml) for 3–5 min^32^,^33^,^34^,^35^. The dissociated cells were then transferred to a dish for recording. The cochlear supporting cells and hair cells can be unambiguously identified under microscope by their own morphological shapes (Fig. S1, and also see ref. 26,32,33). The dissociated supporting cells also retained good gap junctional coupling^33^,^34^,^35^.

To assess intercellular permeation of gap junctions to miRNAs, a fluorescence-tagged miRNA (miR-F), which is constructed by a 25 nt miRNA (5′-CCT CTT ACC TCA GTT ACA ATT TATA-3′) labeled with carboxyfluorescein on its 3′ end (Gene Tools, Inc. OR), was used. This miR-F was proven to not be hybridized or degraded and also had no fluorescent tag removal in the cytoplasm^36^,^37^.

For dye injection to assess intercellular diffusion, a group or pair of cochlear supporting cells was selected. Intracellular injection was performed by patch clamp recording under the whole-cell configuration^26^. The patch pipette was 1.5–2 μm in tip diameter and filled with the normal intracellular solution (KCl 140, EGTA 5, and HEPES 10 in mM, pH 7.2 and 300 mOsm) with 100 μM miR-F. The holding voltage was set at −40 mV. Gap junctional coupling between cells was continuously monitored by input capacitance (*C*_*in*_), which was recorded online at 1–3 Hz and calculated from the transient charge elicited by small (−10 mV) test pulses at the holding potential^33^,^34^. The diffusion was captured with a CCD camera under a fluorescence microscope (Nickon, TE300) as we previously reported^26^.

In some cases, cationic dye ethidium bromide (EB, 0.1 mM) was also used and mixed with miR-F for injection. EB can distinctly identify the transjunctional-diffused cells and clearly demonstrate transjunctional transport, because it can bind to DNAs labeling cell nuclei showing bright fluorescence.

### Immunofluorescent staining

The immunofluorescent staining was performed as previously reported^21^,^38^. The cochlear section or culture cells were fixed with 4% paraformaldehyde for 30 min and washed out with PBS. After 30 min of incubation in a blocking solution (10% goat serum and 1% BSA in PBS) with 0.1% Triton X-100, the cochlear section or culture cells were incubated with monoclonal mouse anti-Cx26 (1: 400, Cat#33–5800, Invitrogen) in the blocking solution at 4 ^o^C overnight. For double immunofluorescent staining for Cx26 and Cx30, polyclonal rabbit anti-Cx30 (1:400, Cat#71–2200, Invitrogen), or polyclonal goat anti-prestin (1:50, Cat# sc-22694, Santa Cruz Biotech Inc, CA) was used. After being washed with PBS, the section or cells were incubated with corresponding Alexa Fluor 488- or 568-conjugated goat anti-mouse IgG and Alexa Fluor 568-conjugated goat anti-rabbit IgG (1:500, Molecular Probes) in the blocking solution at room temperature (23 ^o^C) for 1 hr. In some cases, following the 2^nd^ antibody incubation, the section or cells were stained by 4′, 6-diamidino-2-phenylindole (DAPI, 0.1 mg/ml, D1306; Molecular Probes) for ~15–20 min to visualize cell nuclei. After completely washing out with PBS, the section or cells were mounted with a fluorescence mounting medium (H-1000, Vector Lab, CA) and observed under a fluorescence microscope (Nickon, T2000) or a confocal microscope (Leica TCS SP2). The fluorescent image was saved in the TIFF format and assembled in Photoshop (Adobe Systems, CA) for presentation.

### miRNA extraction and quantitative PCR measurement

The cochlear sensory epithelia were freshly isolated as described above and miRNAs were extracted by mirVana miRNA Isolation Kit (AM1560, Ambion, USA) following manufacturer’s instructions. The purity and quantity of miRNA was determined by a NanoDrop ND-1000 Spectrophotometer (NanoDrop Technologies, Inc., Rockland, DE). Then, miRNAs were converted to cDNA using TaqMan^®^ MicroRNA Reverse Transcription Kit (#4366596, Applied Biosystems, CA, USA) with corresponding mouse-specific miRNA reverse transcription templates according to manufacturer’s instructions and measured by use of MyiQ real-time PCR detection system (Bio-Rad Laboratories) with TaqMan^®^ MicroRNA Assay (Applied Biosystems, CA, USA). An internal standard U6 snRNA (#001973, Applied Biosystems, CA) was used as an internal control. The relative quantity of miRNA expression was calculated from the standard curve^39^ and normalized to the amount of the internal standard U6 snRNA.

### Data analysis

Data were expressed as mean ± s.e.m. and plotted by SigmaPlot (SPSS Inc. Chicago, IL). The statistical analyses were performed by SPSS v18.0 (SPSS Inc. Chicago, IL) using one-way ANOVA with a Bonferroni correction.

## Additional Information

**How to cite this article**: Zhu, Y. *et al.* Connexin26 gap junction mediates miRNA intercellular genetic communication in the cochlea and is required for inner ear development. *Sci. Rep.* **5**, 15647; doi: 10.1038/srep15647 (2015).

## Supplementary Material

Supplementary Information
