# Supplementary material for: Connexin26 gap junction mediates miRNA intercellular genetic communication in the cochlea and is required for inner ear development
Source: Sci Rep. 2015 Oct 22;5:15647. doi: 10.1038/srep15647 (PMC4614881; doi:10.1038/srep15647)
Supplement: Supplementary Information [file srep15647-s1.pdf]

Supplementary figures:

**Connexin26 gap junction mediates miRNA intercellular genetic communication in the cochlea and is required for inner ear development**

Yan Zhu, Liang Zong, Ling Mei, Hong-Bo Zhao

Department of Otolaryngology, University of Kentucky Medical Center, 800 Rose Street, Lexington, Kentucky, USA, 40536

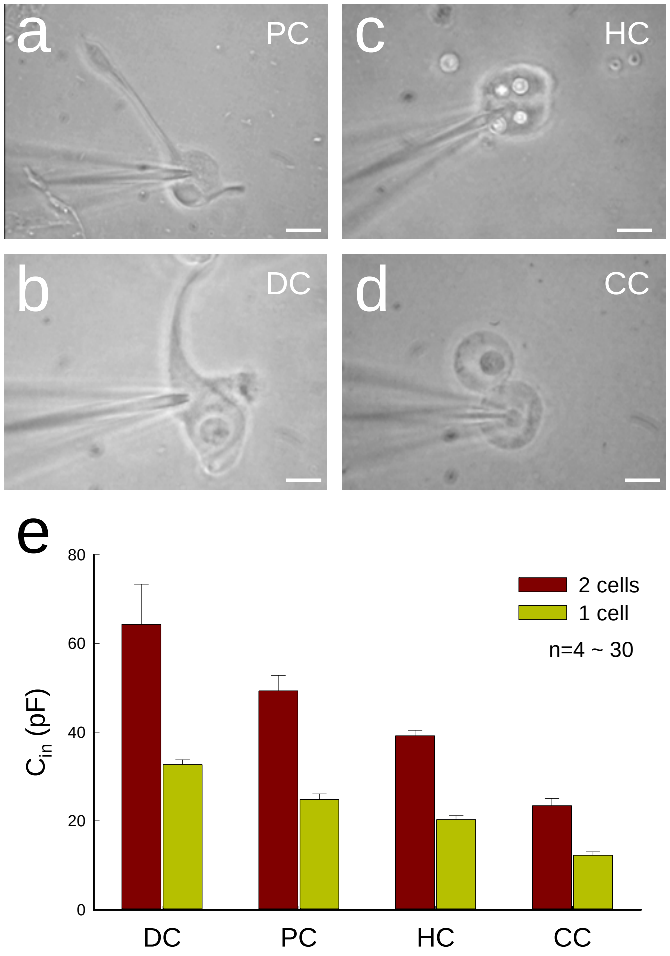


# Supplementary Figure 1

Isolated cochlear supporting cells and input capacitance.

**a-d:** Isolated cochlear supporting cells under patch recording. PC: Pillar cell, DC: Deiters cell, HC: Hensen cell, CC: Claudius cell. Scale bars: 10 µm.

**e:** Input capacitance ($C_{in}$) recorded from single and paired cochlear supporting cells in mice. $C_{in}$ of 2-coupled cochlear supporting cells is double of $C_{in}$ in single cochlear supporting cell.

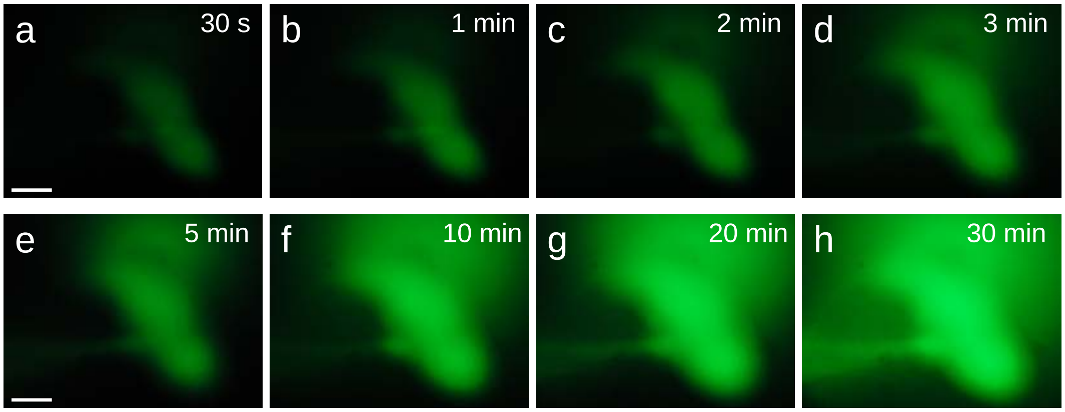


## Supplementary Figure 2

Time-lapse recording of diffusion of fluorescence-tagged miRNA injected at a Deiters cell in the cochlear sensory epithelium. Scale bars: 10 µm.
